# A Matter of Taste: Roles of Taste Preference on Performance and Psychological Responses during Anaerobic Exercise

**DOI:** 10.3390/ijerph20043730

**Published:** 2023-02-20

**Authors:** Davis B. Henry, Anna L. Pemberton, Rebecca R. Rogers, Christopher G. Ballmann

**Affiliations:** Department of Kinesiology, Samford University, 800 Lakeshore Dr. Birmingham, Homewood, AL 35229, USA

**Keywords:** bitter, sour, sweet, sprint, motivation

## Abstract

Various tastes including sweet, bitter, and sour have been shown to differentially influence physiological and psychological processes. Furthermore, ingestion of bitter and sweet solutions has been shown to acutely enhance exercise performance. However, the taste is highly individualized, and it is unclear if preference influences the ergogenic potential of taste. The purpose of this study was to investigate the effects of preferred and non-preferred drink tastes on anaerobic performance and psychological responses thereof. Physically active females participated in two counterbalanced sprint trials each with a different condition: (1) non-preferred taste (NPT), (2) Preferred taste (PT). Participants self-reported taste preferences (sweet, sour, bitter) with the highest-ranked taste being used for the PT condition and the lowest-ranked for NPT. For each visit, participants completed a 15 s Wingate Anaerobic Test (WAnT) prior to (PRE) ingestion of ~20 mL of their NP or PREF taste. Following ingestion, participants completed 2 min of active recovery, rated their taste preference of the solution, and completed another 15 s WAnT. The rate of perceived exertion (RPE), motivation, and enjoyment were measured through a visual analog scale following each WAnT. Anaerobic performance measures and heart rate (HR) were also obtained at the succession of each WAnT. Findings revealed no differences between taste conditions for mean power (*p* = 0.455), peak power (*p* = 0.824), or HR (*p* = 0.847). RPE was significantly lower with PT versus NPT (*p* = 0.006). Exercise enjoyment (*p* = 0.022) was higher with PT compared to NPT. NPT resulted in worse motivation compared to PRE (*p* = 0.001) while no changes were observed between PT and PRE (*p* = 0.197). These findings suggest that preferred drink taste may not enhance acute performance but improves psychological responses to maximal anaerobic exercise which may have implications for improving exercise training and adherence.

## 1. Introduction

The gustatory system allows for the perception of taste in 5 different modalities: sweet, sour, bitter, salty, and umami. It is largely comprised of a myriad of oral receptors that may be directly or indirectly linked to higher brain centers. Indeed, various taste sensations have been shown to differentially activate various parts of the brain (i.e., orbitofrontal cortex, amygdala, etc.) responsible for emotional states, stress responses, and somatic behavior [1,2]. In turn, many investigations have shown that taste sensations may alter both psychological and performance outcomes during exercise [3,4,5]. However, much less is known as to how the preference of taste sensations may influence its ergogenic potential. 

Of the different taste modalities, sweet, sour, and bitter have been the most researched in the context of exercise. Sweet-tasting carbohydrate mouth rinses (i.e., glucose, and fructose) and ingested solutions have been reported to improve both aerobic and anaerobic performance, albeit large amounts of heterogeneity between studies have been noted [6]. Interestingly, flavorless carbohydrate solutions have been suggested to be less efficacious than sweet-tasting counterparts [6]. This may suggest that the presence of taste, not solely carbohydrate content, mediates the ergogenic effects of taste sensation. Although evidence has suggested sweet and sour tastes result in similar brain activation [7], sour tastes (i.e., lemon juice, citric acid) have not been reported to improve performance although there is little research in this area [8]. Still, others have shown that sour tastes may result in greater muscle activation and electromyography signals compared to tasteless solutions [9]. Bitter tastes have been linked to improved performance in high-intensity exercise and sprints [4]. Presumably, this is mediated by the activation of areas of the brain (i.e., insular cortex, amygdala) responsible for emotion and motor control [4,5]. While there are numerous other investigations reinforcing these findings, almost all studies to date have used a single taste modality or pre-determined flavor which does not account for individual preferences of taste.

In addition to the taste modality itself, whether or not the individual finds the sensation favorable or unfavorable largely determines physiological and psychological alterations. Preferred tastes have been shown to result in marked increases in activation of the ventral striatum, ventral pallidum, and amygdala [10]. Together, these may interact as regulatory centers for reward-induced changes in behavior. Indeed, preferred tastes have been shown to potently activate the nucleus accumbens (NAc) which is central to reward-driven pathways and the release of dopamine [11]. This in turn may increase subjective feelings of well-being, motivation, and enjoyment [12]. Aversive tastes may also alter physiological and psychological processes. Aversive stimuli, including taste, have been shown to lower motor cortex activation [13]. Lower activation of the motor cortex may in turn lead to poorer performance and muscular force output. Psychologically, unpleasant tastes may invoke less desirable hedonic responses and negatively alter affective states [14,15]. Less is known if eliciting these processes influences the response to exercise. 

To date, little evidence exists as to whether taste preference influences exercise performance. However, Roitman et al. showed that there is a positive relationship between taste-induced NAc activation and motor output which could suggest possible ergogenic effects of preferred tastes [16]. Conversely, unpleasant tastes have been shown to induce sympathetic nervous system activation but did not influence neuromuscular performance [17]. Given the purported physiological and psychological changes with varying perceptions of taste, it is plausible that taste preference may independently affect exercise performance aside from taste modality. Thus, the purpose of this brief report was to investigate the effects of preferred versus non-preferred taste on anaerobic sprint performance and psychological responses thereof. We hypothesized that choosing a preferred drink taste of bitterness, sweetness, or sourness would result in increased sprint performance, exercise enjoyment, and motivation, and a decreased rate of perceived exertion (RPE) versus non-preferred drink taste in college-aged females.

## 2. Materials and Methods

### 2.1. Study Design

Using a blinded, counterbalanced, crossover approach, young adult females completed two visits each with a different experimental condition: Preferred taste (PT) or non-preferred taste (NPT). During each visit, participants completed a pre-taste (PRE) Wingate Anerobic Test (WAnT) to establish baseline performance followed by 2 min of active recovery. Following this, participants ingested their PT or NPT and then completed a second WAnT. Performance variables, heart rate (HR), rate of perceived exertion (RPE), motivation, and enjoyment were recorded after each WAnT. Comparisons were drawn between PRE, PT, and NPT with each trial separated by a 48-h washout period.

### 2.2. Participants

A convenience sample of healthy physically active females (n = 12; 20.6 yrs ± 0.5, 62.7 kg ± 7.9, 165.0 cm ± 7.6) were recruited from Samford University and the surrounding Birmingham, AL, USA area. The sample size was chosen to be comparable to previous investigations on taste and exercise performance [3,5,6,18]. Physically active was defined as accruing 150 min/wk of moderate to vigorous physical activity/exercise [19]. Further inclusion criteria included being free from a lower-body injury in the past six months, having no history of taste disorders, and having no major medical conditions (i.e., cardiovascular, metabolic, renal diseases). Screening for exercise suitability was accomplished using a physical activity readiness questionnaire (PAR-Q) and was mandatory prior to involvement in exercise. Participants were asked to refrain from alcohol, caffeine, and nicotine for a minimum of 6 h before testing [20,21]. Written and informed consent was obtained from each participant prior to data collection. All experimental procedures were conducted in accordance with the Declaration of Helsinki and approved by the Samford University Institutional Review Board (EXPD-HP-22-SUM-5; June 2022).

### 2.3. Taste Preference and Solutions

To determine taste preference, participants took a survey ranking sweet, sour, and bitter tastes from 1 (most preferred) to 3 (least preferred). Sweet, sour, and bitter tastes were selected as the previous literature has reported positive effects on neuromuscular/exercise performance [3]. To aid in the understanding of selections, common food/drink examples were provided for each taste for reference (see below). Percentage breakdowns of how participants chose PT and NPT are also shown:

- Sweet: Maple syrup, Honey, Watermelon
  ○ Rating of PT: 91%
  ○ Rating of NPT: 0%
- Sour: Lemon, Lime, Oranges
  ○ Percent PT: 9%
  ○ Percent NPT: 17%
- Bitter: Coffee, Dark chocolate, Grapefruit
  ○ Percent PT: 0%
  ○ Percent NPT: 83.3%

All solution concentrations were adapted from previous taste investigations [22,23,24]. For the sour condition, the tasting solution was made by dissolving food-grade citric acid anhydrous (Milliard, NJ, USA) into 20 mL of water to form a 0.5% solution [24]. For the bitter condition, the solution was made using a quinine liquid extract (2 mM stock; HERBAMAMA LLC, Las Vegas, NV, USA) into 20 mL of water to make a 0.1% quinine solution [23]. Lastly, the sweet solution was made by dissolving sucralose (Splenda; Heartland Food Products; Carmel, IN, USA) into 20 mL of water to make a 1% sucralose solution. During the active recovery period between WAnTs, participants swished 20 mL of their PT and NPT solution in their mouths for 20 s and then ingested it [23]. To confirm differences in preference between solutions, participants were then asked to rate the taste of each solution on a scale of 0–100 using a visual analog scale where 0 indicated “worst taste ever” and 100 indicated “best taste ever”. This was completed immediately prior to the second WAnT. Although participants were not fully blinded due to the subjective nature of the treatment, they were unaware of the treatment order prior to participation. Participants were not aware of any experimental hypotheses related to their tastes.

### 2.4. Procedures

After height and body mass were documented, participants were first outfitted with a chest strap HR monitor (Lake Success, NY USA). Participants then completed a 5-min standardized warm-up where they pedaled at 50 watts on a mechanically braked cycle ergometer with a pedal rate synchronized to a metronome at 60 bpm (Monark, Varberg, Sweden). For the testing portion, participants completed 2 × 15 s WAnTs on an electronically braked cycle ergometer (Velotron, Racermate Inc., Seattle, WA, USA) as similarly described by our lab [21,25,26,27]. Participants began each WAnT with a 10-s lead-in phase to allow for the attainment of maximal pedal rate. Resistance was then immediately applied at 7.5% of the participant’s body mass and participants pedaled maximally for 15-s. The WAnTs were separated by 2 min of active recovery. Since it has been suggested that varying tastes may be more ergogenic under fatigued or stressed conditions [3], participants completed their first WAnT on each visit prior to taste administration (PRE) to establish this. Both PRE WAnTs were averaged for analysis to serve as a baseline and to account for day-to-day variation in performance. During the active recovery period and immediately prior to the second WAnT, participants ingested their PT or NPT solution as subsequently described and then repeated the WAnT procedure. After each WAnT, power measurements, HR, RPE (1–10 scale), and psychological measures were collected. Subjective feelings of motivation and enjoyment were used as psychological variables and measured using a visual analog scale as previously described by our lab and others [25,28,29,30]. Briefly, participants marked their subjective feelings on a 100 mm line whereby 0 indicated the absence of the feeling and 100 indicated the strongest feeling. All participants were verbally encouraged during the testing. 

### 2.5. Statistical Analysis

Data analysis was completed using Jamovi software (Version 0.9; Sydney, Australia). Confirmation of data normality was conducted using a Shapiro-Wilk test. To determine differences in taste rating, means were compared and analyzed using a pairwise *t*-test. All other variables were analyzed using a 1 × 3 repeated measures ANOVA [test × condition] to detect the main effects. A Bonnferroni-Holm posthoc test was used to determine differences in means for significant main effects. Estimates of effect size for main effects were calculated using eta squared (η^2^) and interpreted as: 0.01—small; 0.06—medium; ≥0.14—large [31,32]. Effect sizes between means for t-tests were calculated via Cohen’s d (d) and interpreted as: 0.2—small; 0.5—moderate; 0.8—large [31,32]. All data are presented at mean ± standard deviation (SD). Significance was set at *p* ≤ 0.05 *a priori*.

## 3. Results

### 3.1. Taste Preference, Mean, and Peak Power

**Figure 1:**
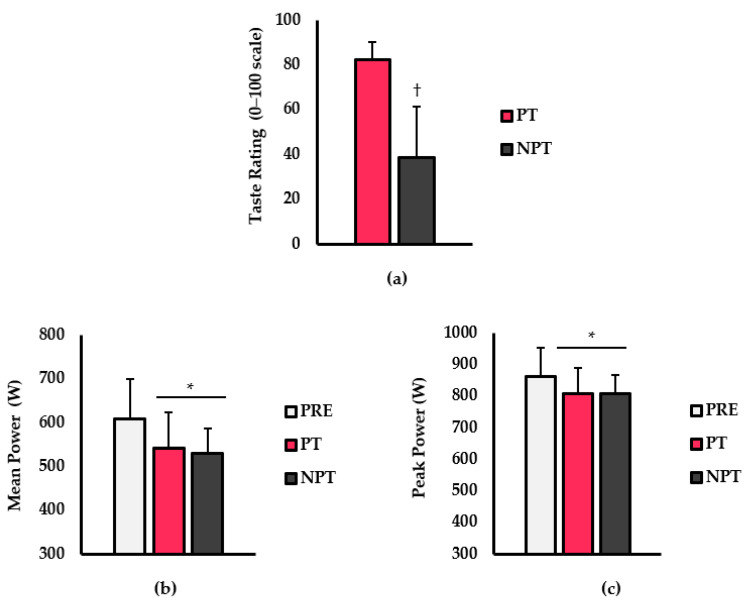
Differences in (**a**) Taste rating (0–100 scale), (**b**) Mean power (watts_;_ W), and (**c**) Peak power (watts; W) between pre-taste (PRE; light grey bars), preferred taste (PT; pink), and non-preferred taste (NPT; dark grey) conditions. Data are presented as mean ± SD. † indicates significantly different from PT (*p* < 0.05). * indicates significantly different from PRE (*p* < 0.05).

Taste ratings, mean power, and peak power are shown in Figure 1. For taste rating (0–100 scale; Figure 1a), participants rated the PT significantly more favorable than the NP (*p* < 0.001; d = 2.8). For mean power (watts; Figure 1b), there was a main effect for treatment (*p* < 0.001; η^2^ = 0.180), whereby performance in the PT (*p* < 0.001) and NPT (*p* = 0.003) were significantly lower than PRE. No differences existed between PT and NPT (*p* = 0.455). For peak power (watts; Figure 1c), a significant main effect for treatment also existed (*p* < 0.001; η^2^ = 0.019). Peak power was significantly higher during PRE versus PT (*p* = 0.003) and NPT (*p* = 0.007). However, no differences between PT and NPT were observed (*p* = 0.824).

### 3.2. HR, RPE, Motivation, Enjoyment

**Figure 2:**
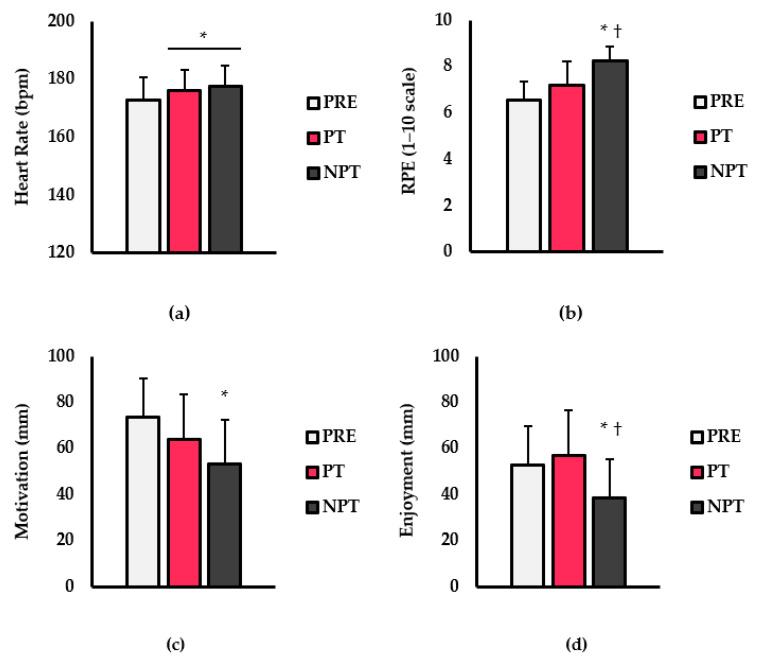
Differences in (**a**) Heart rate (HR; bpm), (**b**) Rate of perceived exertion (RPE; 1–10 scale), (**c**) Motivation (mm), and (**d**) Enjoyment (mm) between pre-taste (PRE; light grey bars), preferred taste (PT; pink), and non-preferred taste (NPT; dark grey) conditions. Data are presented as mean ± SD. † indicates significantly different from PT (*p* < 0.05). * indicates a significantly different from PRE (*p* < 0.05).

Results for HR, RPE, motivation, and enjoyment are shown in Figure 2. For HR (bpm; Figure 2a), there was a main effect for treatment (*p* < 0.001; η^2^ = 0.119). HR was significantly lower during PRE than PT (*p* < 0.001) and NPT (*p* < 0.001). No differences existed between PT and NPT (*p* = 0.847). For RPE (1–10 scale; Figure 2b), there was a main effect for treatment (*p* < 0.001; η^2^ = 0.436). RPE was significantly higher with NPT compared to PRE (*p* < 0.001) and PT (*p* = 0.006). No differences were noted between PT and PRE (*p* = 0.115). Analysis of motivation (mm; Figure 2c) revealed a main effect for treatment (*p* < 0.001; η^2^ = 0.174). The motivation was significantly lower with the NPT compared to PRE (*p* = 0.001). No other differences were noted. Lastly, enjoyment (mm; Figure 2d) was lower with NPT compared to PRE (*p* = 0.005) and PT (*p* = 0.045). No differences were noted between PRE and PT (*p* = 0.728).

## 4. Discussion

The influence of particular tastes on exercise performance has been widely studied in various contexts [3,5]. Of the available evidence currently, most studies have reported performance-enhancing effects during power-based exercise with sweet and bitter taste sensations [3,5,18]. While promising, responses to taste are highly individualized, and little to no studies to date have accounted for preference and how it might influence results differently. Findings from the current investigation show that power output was not enhanced regardless of taste preference. However, RPE was significantly higher, and enjoyment was significantly lower with NPT versus PT. The motivation was also lower with NPT versus PRE while motivation was similar between PRE and PT conditions. Although more study is warranted, these findings suggest a prominent role for taste preference in psychological responses but not performance during power-based activities. 

The bulk of participants chose sweet as their PT while bitter was rated as the NPT by the majority of individuals. Confirmation of taste preference was obtained by subjective rating and as expected, PT (sweet for most responses) was rated more favorably than NPT (bitter for most responses). Indeed, innate preferences for sweet tastes may be heightened in childhood and can last through early adulthood [33,34,35]. Despite this, no differences in mean or peak power were noted between conditions regardless of taste preference which is in opposition to previous work investigating sweet taste and performance. For example, a systematic review conducted by Silva et al. reported that sweet-tasting carbohydrate mouth rinses were ergogenic in power-based and explosive exercises [6]. Differences may in part be due to the absence of caloric content in the sweet condition as sucralose was used instead of sucrose/glucose as used in previous investigations. Previous research has shown that sucrose elicits great neural activation compared to non-caloric sweeteners [36]. Furthermore, the induction of reward pathways and dopamine release is less pronounced with sucralose compared to sucrose which may be due to additive stimulation by oral sucrose receptors [36]. Thus, the lack of performance enhancement in the current investigation with PT may have been due to the absence of carbohydrates resulting in diminished neural stimulation. However, the interaction between taste preferences and carbohydrate content on exercise performance remains unclear and should be the topic of future studies. Lack of performance enhancement may also be due to the exercise time of day. Clarke et al. showed that carbohydrate mouth rinse improved vertical jump and sprinting performance during morning hours [18]. While testing times were kept consistent between participants in the present investigation, direct manipulation of the time of testing was not carried out. Since it has been generally shown that power-based performance suffers during morning times [37,38], thresholds for performance improvement may be lower which may have not been fully realized given the current study design. Although speculative, the lack of improvements in power output with PT may be related to a lower opportunity for performance enhancement as various testing times were used. Direct testing of taste preference and diurnal fluctuations in performance are needed in order to understand optimal timeframes for which taste may exert the greatest benefits. 

Although no changes in performance between taste preferences were noted, taste preference appears to potently influence psychological responses to exercise. More specifically, NPT resulted in greater perturbations of RPE, motivation, and enjoyment. Noxious stimuli, such as unpleasant taste, result in more pronounced sympathomodulation and affective responses [39]. Furthermore, noxious stimuli alter nociception and can result in hyperalgesia [40]. While unknown from the present data alone, increases in sympathetic activation and heightened pain responses may be responsible for psychological changes from NPT. Higher pain perceptions have been linked to higher RPE values [41]. Furthermore, unpleasant tastes may result in potentiated pain responses [42,43]. It is plausible that the NPT resulted in greater exercise-induced hyperalgesia thereby resulting in increases in RPE. Similar mechanisms may be responsible for the decreases in motivation and enjoyment with NPT. Pain has been described to alter motivation and enjoyment levels through mesocorticolimbic pathways [44]. Independent or in concert with this, aversive tastes alter emotion state through the pregenual cingulate which is thought to partially control mood and affective state [45]. While not confirmed, the lower enjoyment with NPT may have manifested in alterations in an emotional state. The same brain regions have been implicated to be responsible for motivation and task preparation [46]. However, the relationship between exercise motivation and enjoyment in the context of taste is unclear leaving the precise contributions to changes in psychological outcomes undetermined. Future studies should look to determine how to taste preference modulates psychological variables during exercise independently and synergistically. 

Although this brief report presents novel information regarding taste preference and exercise performance, there were limitations that should be acknowledged. First, the sample size was small and homogenous leaving the need for larger and more diverse samples to identify precise contributions of taste preference to performance. In particular, more diverse preferences of taste will be needed to make these results more generalizable. Only sprint exercise was employed in the current design. It is feasible that taste preference may influence other modalities of exercise and should be the subject of future investigations. For the psychological analysis, only a single item measurement was used which may not encompass all nuances needed for comprehensive analysis. Future studies using validated multi-item analysis are warranted. It remains generally speculative as to what the exact mechanisms are responsible for the psychological changes currently seen as the present study design did not permit mechanistic exploration. Future studies should use more detailed psychophysiological measurements in order to understand current phenomena. Furthermore, only three out of the five taste modalities were included as choices for preference. This was based on prior evidence suggesting sweet, sour, and bitter tastes possess the largest ergogenic potential [3,5]. We cannot rule out the possibility of different outcomes had all five taste modalities been included.

## 5. Conclusions

In conclusion, taste preference does not appear to alter power output during sprinting. However, NPT resulted in unfavorable changes in psychological variables such as RPE, motivation, and enjoyment. From a practical standpoint, athletes and recreational exercisers regularly consume flavored pre- or post-workout supplements. Anecdotally, many individuals may sacrifice the taste of these products for ingredient profiles that meet their liking or are more cost-effective. Current findings suggest that compromising on taste preference may result in unfavorable psychological outcomes during exercise. Uncomplimentary changes in RPE, motivation, and enjoyment may lead to lower inter-session exercise adherence potentially altering training intensity. Despite the small sample size, the current study suggests individuals should attempt to maintain their taste preferences as much as possible to avoid negative psychological responses to exercise. 

## Data Availability

All data are freely available within this manuscript.
